# Applicability of Metal Nanoparticles in the Detection and Monitoring of Hepatitis B Virus Infection

**DOI:** 10.3390/v9070193

**Published:** 2017-07-21

**Authors:** Maxim Shevtsov, Lili Zhao, Ulrike Protzer, Maarten A. A. van de Klundert

**Affiliations:** 1 Klinikum rechts der Isar, Technischen Universität München (TUM), Ismaniger Str. 22, 81675 Munich, Germany; 2 Institute of Cytology of the Russian Academy of Sciences (RAS), Tikhoretsky Ave., 4, 194064 St. Petersburg, Russia; 3 Institute of Virology, Technische Universität München/Helmholtz Zentrum München—German Center for Environmental Health, Trogerstr. 30, 81675 Munich, Germany; lili.zhao@tum.de (L.Z.); protzer@tum.de (U.P.); maarten.van-de-klundert@tum.de (M.A.A.v.d.K.)

**Keywords:** hepatitis B, virus, magnetic nanoparticles, gold nanoparticles, biosensor

## Abstract

Chronic infection with the hepatitis B virus (HBV) can lead to liver failure and can cause liver cirrhosis and hepatocellular carcinoma (HCC). Reliable means for detecting and monitoring HBV infection are essential to identify patients in need of therapy and to prevent HBV transmission. Nanomaterials with defined electrical, optical, and mechanical properties have been developed to detect and quantify viral antigens. In this review, we discuss the challenges in applying nanoparticles to HBV antigen detection and in realizing the bio-analytical potential of such nanoparticles. We discuss recent developments in generating detection platforms based on gold and iron oxide nanoparticles. Such platforms increase biological material detection efficiency by the targeted capture and concentration of HBV antigens, but the unique properties of nanoparticles can also be exploited for direct, sensitive, and specific antigen detection. We discuss several studies that show that nanomaterial-based platforms enable ultrasensitive HBV antigen detection.

## 1. Introduction

Infections with the hepatitis B virus (HBV) often become chronic, especially when people are infected at a young age. Chronic HBV infection is strongly associated with the development of liver diseases such as liver cirrhosis and hepatocellular carcinoma, resulting in about one million deaths each year [1,2,3]. Although currently no curative therapy for chronic HBV infection is available, treatment of patients with nucleotide analogs or interferon can suppress viral replication and reduce the risk of developing end-stage liver disease.

Several physico-chemical and biochemical methods have been developed to diagnose and quantify HBV infection [4,5,6,7,8]. In most infected patients, HBV DNA can be detected by PCR and secreted HBV antigens, such as the envelope (hepatitis B surface antigen, HBsAg) and core proteins [4,8], can be detected by Enzyme-Linked ImmunoSorbent Assay (ELISA) [5]. Although PCR and ELISA-based methods have proven to have a good specificity, they are not always sensitive enough to detect HBV antigens in patient samples. For instance, in occult HBV infection, HBV DNA can be detected in patient serum in the absence of detectable HBsAg. Notably, occultly infected patients are still at an increased risk of developing HBV infection-related liver disease and may benefit from therapy. In addition, in blood transfusion practice, the detection limits of currently available standardised tests for HBV antigens can cause safety risks, especially because blood samples are often pooled before testing to reduce testing costs.

Nanoparticles have been developed for various applications in the treatment and imaging of liver diseases [9]. Typically, such nanoparticles are coated with biological components (e.g., antibodies, oligonucleotides, aptamers etc.) that grant them a specificity to interact with a specific protein or DNA fragment. For example, gold particles coated with antibodies specific to certain proteins can be used to localise specific proteins at a subcellular level by electron microscopy. Nano-sized materials combined with biomolecules can contribute to the improvement of bio-analytical methods in terms of sensitivity and specificity. With the development of nanotechnology, various nanoparticles including e.g., quantum dots [10], carbon nanotubes [11] and nanowires [12] were applied in bio-analytical assays.

The application of nanoparticle-based detection methods may provide a more sensitive alternative for the diagnosis and monitoring of viral infections. In this review, we provide an overview of recent advances in the development of diagnostic tools with specific focus on the application of gold and iron oxide nanoparticles that have gained much attraction in recent years [13,14,15] in the detection and quantification of HBV infection.

## 2. Gold Nanoparticles

Gold nanoparticles (AuNPs) have often been used as carriers for various biomedical applications due to their biocompatibility, their optical and electronic properties and because they are relatively easy to manufacture [16]. AuNPs can be functionalised with various biological macromolecules, such as antibodies, oligonucleotides and aptamers, to detect a variety of (bio) molecules [17]. For instance, antibody-coated AuNPs can be used to stain substrates for electron microscopy in order to determine the (sub) cellular localisation of (viral) proteins [18,19].

Over the last few decades, various methods have been developed that employ the unique physical properties of AuNPs to detect and quantify biological molecules in samples. These methods have the potential to improve the sensitivity, ease of operation and applicability of HBV detection [20].

For instance, Wu et al. employed AuNPs dually labelled with anti-HBsAg antibodies and human alpha-thrombin (HAT, an enzyme that can convert a bisamide substrate into a fluorescent reaction product) [21]. These AuNPs were used to detect HBsAg bound to anti-HBsAg coated on a conventional ELISA plate by enhanced fluorescence enzyme-linked immunosorbent assay (FELISA). Under optimal conditions (HBsAg was dissolved in phosphate-buffered saline (PBS)), this method allowed the detection of HBsAg concentrations of 5 × 10^−4^ IU/mL, which is about 10^4^ times lower than the detection limit of other fluorescence-based methods and 10^6^ times lower than those of the conventional ELISA [21].

### 2.1. Detection of Hepatitis B Virus Antigens by Gold Nanoparticles Surface Plasmon Resonance

One of the unique physical properties of the AuNPs is their specific optical behaviour when exposed to electromagnetic radiation. This causes an oscillation of the electrons, called surface plasmon resonance (SPR), which depends on the size and shape of the nanoparticle and (the dielectric constant of) its environment [22]. Consequently, interactions between molecules covering the AuNP and molecules in the environment cause changes in the SPR frequencies of the AuNP that can be detected and used to quantify specific biological molecules in their environment. Several bio-analytical applications based on AuNP SPR have been reported [23,24,25]. AuNP SPR has been used to quantify HBsAg in blood, serum and plasma by directly measuring the shift in the SPR peak of anti-HBsAg coated AuNP [26]. The authors were able to detect HBsAg concentrations of 0.1 IU/mL [26].

Interestingly, changes in the SPR of AuNPs can be in the visible part of the spectrum, allowing the determination of a reaction by colour shifts in the visible spectrum. Typically, for this format, AuNPs are immobilised on paper strips and used to detect PCR-amplified pathogen DNA, which greatly enhances their applicability in resource-poor settings. For example, AuNPs have been combined with inkjet-printed, dye-sensitised TiO_2_ photodetectors as a means of detection to generate colorimetric biosensors with a limit of detection (LOD) of 1 nm DNA [27]. Recently, this method was further enhanced to simultaneously detect two different pathogens in one reaction [28]. Duan et al. recently employed immobilised HBV and hepatitis C virus (HCV) antigens, staphylococcal protein A (SPA)-labelled AuNPs and a silver staining step to increase the optical signal to simultaneously detect antibodies to HBV and HCV antigens [29]. The method was tested on 305 serum samples, of which antigen concentrations were previously determined by ELISA, showing a comparable sensitivity and a LOD of 3 ng/mL antibody [29]. Interestingly, Song et al. used oligonucleotide-directed precipitation of AuNP on a plate carrier to identify tyrosine-methionine-aspartate-aspartate (YMDD) mutations in patient-derived HBV DNA [30]. The release of AuNPs from a carrier can also be monitored by dark-field microscopy. Jang et al. used AuNPs coupled to multiple pathogen-specific oligonucleotides with restriction enzyme specific bridging sequences to simultaneously detect femtomolar amounts of hepatitis A virus (HAV), HBV and human immunodeficiency virus (HIV) cDNA using sequential incubation with different restriction enzymes [31].

### 2.2. Use of Gold Nanoparticles in Electrochemical Detectors

**Figure 1:**
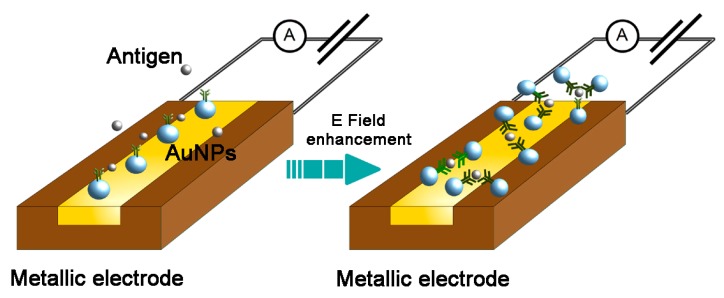
Schematic representation of gold nanoparticles applied in an electrochemical biosensor. E Field: electric field; AuNPs: gold nanoparticles.

The electrochemical features of the AuNPs make them attractive carriers to grant specificity to electrochemical biosensors (Reviewed in [32]). AuNP-based electrochemical biosensors have been designed for DNA [33,34,35] and protein [36] quantification and analysis. The principle of the method is based on the complex formation between oligonucleotide- or antibody-coated AuNPs and specific DNA fragments or proteins at an electrode surface that results in the production of detectable amperometric, potentiometric or impedimetric signals (Figure 1). Notably, the detection of viral DNA does not require a PCR amplification step. Several electrochemical biosensors have been used to detect HBV antigens. Streptavidin-conjugated AuNPs have been combined with a biotin-labeled, HBV DNA-specific DNA probe and applied for the voltammetric detection of HBV DNA with a LOD of 2 × 10^−12^ M viral DNA [37]. Chen et al. designed and tested an impedance biosensor for HBV DNA which had a LOD of 111 copies/mL [35]. Several electrochemical biosensors have been developed to detect HBsAg [36,37,38,39,40,41,42], with sensitivities ranging from 0.358 pg/mL [36] to 1.9 pg/mL.

### 2.3. Gold Nanoparticles-Based Lateral Flow Assay

Because they are easy to operate and do not require reagents or machines to be read out, lateral flow assay (LFA)-based detection methods are often applied in point-of-care diagnostics [20]. Several studies suggest that the main disadvantage of such tests, that of their low sensitivity, can be improved by AuNP-based signal amplification [43]. Kim et al. developed an AuNP-based LFA that could detect 500 ng/mL HBsAg in whole blood, which was comparable to a commercially available HBsAg LFA (Humasis, Anyang, Republic of Korea) [44].

### 2.4. Gold Nanoparticles-Enhanced Raman Spectroscopy

Raman spectroscopy is the analysis of the scattering of low energy electromagnetic radiation by inelastic collision with an analyte [20]. Adsorption or immobilisation of an analyte on AuNPs can greatly (10^6^) enhance the probability of Raman scattering, a phenomenon called surface enhanced Raman spectroscopy (SERS). Intriguingly, SERS-based detection methods have been developed with a sensitivity in the order of single molecules [17,45]. A gold nanostructure SERS-based HBsAg assay was developed, which had a sensitivity of 0.01 IU/mL, a good specificity and a broad linear range [46].

## 3. Magnetic Nanoparticles

Because biological materials lack magnetic behaviour, magnetic nanoparticles (MNPs) can be used to detect specific molecules in biological samples without causing interference with signal detection [47]. MNPs based on iron oxide are one of the most widespread NP formulations applied in biomedical research [48] and have been applied in various electrochemical, optical, piezoelectric and magnetic field sensors [49,50,51,52,53,54,55]. For the synthesis of MNPs, several types of magnetic iron oxides including magnetite (Fe_3_O_4_), hematite (α-Fe_2_O_3_) and maghemite (γ-Fe_2_O_3_ and β-Fe_2_O_3_) are used [56]. As the magnetism of such particles relies on superparamagnetism, they are often referred to as superparamagnetic iron oxide nanoparticles (SPIONs). A widely used and straightforward application of such particles is magnetic-activated cell sorting (MACS), in which specific cells are labelled with antibody-conjugated magnetic particles and subsequently sorted by exposure to a magnetic field. The ease of sorting MNP-bound molecules has also been used to develop HBsAg-specific aptamers that were subsequently used to detect HBsAg by ELISA with a LOD of 0.1 ng/mL [57].

### 3.1. Spin–Spin Relaxation Time-Based Detection Methods

**Figure 2:**
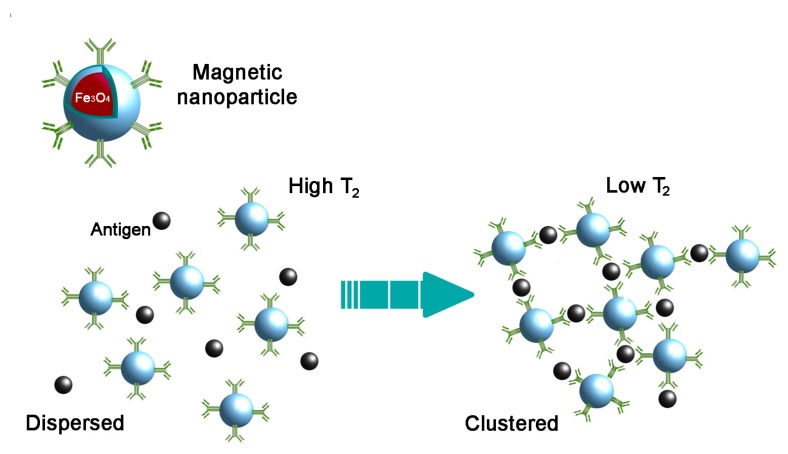
Schematic representation of magnetic nanoparticle (MNP) clustering in the presence of a specific antigen. Following interaction between functionalized MNPs and an antigen, the clustering of MNPs induces a change in the T_2_ relaxation values of the surrounding water molecules, which can be detected by (diagnostic) magnetic resonance.

Most MNP-based antigen detection methods are based on changes in the spin-spin relaxation time (T_2_) of water molecules surrounding an MNP upon the clustering of the MNP induced by a specific target (Figure 2). Changes in T_2_ can be quantified using conventional magnetic resonance imaging (MRI) scanners or nuclear magnetic resonance (NMR) relaxometers. Notably, such devices are becoming increasingly practical to work with (i.e., benchtop format) and sensitive. Recently, Wang et al. demonstrated that, using MNPs and an ultra-low field (ULF) NMR technique, they could detect protein concentrations of 10 pg/mL, below the LOD of conventional ELISA [58]. More recently, chip-based NMR detection systems have been developed which can process multiple microliter volumes samples [54].

### 3.2. Electrochemical Detection

Magnetic nanoparticles can be used for the electrochemical detection of an interaction with a specific ligand, e.g., through direct contact with the electrode, the transfer of electrons generated in redox-reactions, or the formation of a film on the electrode surface [50]. Fatemi et al. used MNPs to capture PCR-amplified HBV DNA and subsequently detect the presence or absence of DNA by cathodic stripping voltammetry [59]. Although this method requires PCR-amplified DNA, it has a good potential for miniaturisation (i.e., lab-on-a-chip) application [59]. Nourani et al. applied anti-HBsAg coated MNPs to capture HBsAg and a horseradish peroxidase (HRP)-labelled secondary antibody to convert aminophenol into electrochemically detectable reaction products, with an LOD of 0.9 pg/mL [59,60]. Magnetic nanoparticles have been applied to capture HBV DNA prior to analysis in a commercially available microfluidic electrophoresis system (Experion, Bio-Rad, Hercules, CA, USA) [61].

The magnetic properties of MNPs have also been used to assemble oligonucleotide-labelled MNPs on an electrode surface by the application of a magnetic field. This electrode was subsequently applied to detect HBV DNA by impedance spectroscopy (an electrochemical technique to characterize film formation on conductive surfaces [62]), with a LOD of 2.5 nm HBV DNA [63].

### 3.3. Lateral Flow Assay

Zhang et al. constructed an MNP-based HBsAg lateral flow assay. Using human serum samples, they demonstrated the LFA strips had an LOD of 5 pg/mL for manual (i.e., naked eye) detection and of 0.1 pg/mL for detection by mechanical analysers [64].

## 4. Quantum Dots

Because of their photochemical stability, quantum dots (QDs) are a promising alternative to organic fluorophores [65]. As such, QDs have been applied to detect HBV DNA [66], anti-HBsAg antibodies [67], HBV mutants [68,69], and HBsAg [70]. Except for the detection of Anti-HBsAg (LOD: 2 pg/mL [67]), the sensitivity of QD-based detection of HBV antigens is below that of other methods.

## 5. Combinations of Different Nanoparticles

Detection methods employing different nanomaterials can increase the effectiveness and applicability by combining the properties of individual nanoparticles [71]. Mashhadizadeh and Talemi [72] combined antisense DNA probes, immobilised on AuNPs and linked to a carbon paste electrode, to measure the competition between target (HBV) DNA and MNPs by assessing the change in interfacial charge transfer resistance (R*_CT_*). The LOD of this method was 3.1 (±0.1) × 10^−13^ M HBV DNA, considerably lower than detection methods employing either nanoparticle alone (Table 1).

Gold nanoparticles and magnetic nanoparticles have also been combined for the colorimetric quantification of target DNA or RNA. Briefly, AuNPs were labelled with oligonucleotides that, upon hybridisation to a specific target RNA or DNA sequence, undergo a click-chemistry reaction which could be amplified by thermal cycling. Subsequently, AuNPs were precipitated using MNPs specific to the reaction product. The (visible) change in the reaction supernatant SPR (i.e., colour) served as a readout [73]. This technique was able to detect several copies of target DNA, comparable to PCR-based methods. The method was not validated for detecting HBV DNA. Alizadeh et al. designed an electrochemical HBsAg immunosensor by assembling anti-HBsAg coated MNPs on an electrode as a supporting matrix, and peroxidase-labelled AuNPs were used to generate a voltametrically detectable signal [77], with a LOD of 0.19 pg/mL HBsAg. Shen et al. combined AuNPs with MNPs to detect HBsAg by anodic stripping voltammetry, with a LOD of 87 pg/mL [75].

Gold nanoparticleshave also been combined with multi-colour QDs to simultaneously detect HBV and HCV DNA. QDs of different colours were coated with HBV DNA or HCV cDNA specific probes and captured on glassy carbon electrodes. These were incubated with target DNA, which prevented the binding of target DNA coated AuNPs in a dose-dependent manner. Subsequently, target DNA concentrations were determined by the quenching of QD electrochemoluminescence by AuNPs [76]. By employing these methods, the authors could detect HBV DNA with concentrations as low as 8.2 × 10^−14^ M in human serum without PCR amplification.

Oligo-labelled AuNPs and MNPs have been applied to capture HBV DNA, concentrate and purify it using a magnet and measure silver nanoparticle (AgNP) amplified voltammetric signals on a device consisting of electrodes and folded paper [78]. Although, with a LOD of 85 pM HBV DNA, the device was not the most sensitive, its application is virtually reagent-free and the disposable device can be assembled for around 0.36 U.S. dollars, making it conceptually interesting for point-of-care (POC) HBsAg determination in low-resource settings [78].

## 6. Conclusions

Adequate means to detect HBV antigens in serum samples are essential for providing adequate individual patient care, but also in guaranteeing the safety of transfusable human blood products [6,7,8]. Numerous studies have shown that the application of nanoparticles can greatly improve the sensitivity and applicability of diagnostic methods. The possibility of detecting NP-associated antigens by SPR, electrochemistry and NMR has been demonstrated for HBV antigens and offers interesting perspectives for miniaturisation. Further (commercial) development and the validation of NP-based detection techniques could be of great use in increasing the safety and decreasing the costs of testing in blood transfusion product production. The signal enhancement of LFA-based detection strips by NPs may increase their applicability, especially in POC HBV testing. Combinations of such techniques can be used to generate cheap, easy to operate and reagent-free HBsAg tests. If the sensitivity of such tests could be improved, they may be a favourable alternative for POC HBV status determination in resource-poor settings [27].

## Figures and Tables

**Table 1:** Overview of different nanoparticle-based detection methods and their detection limits.

Method	Nanoparticle Use	Detected Antigen	Detection Method	Lower Limit of Detection	Substrates Tested	Ref.
Conventional methods	-	Anti-HBsAg	ELISA		Plasma, serum	[73]
	-	HBsAg	ELISA	0.5 IU/mL	Plasma, serum	[19]
	-	HBV DNA	PCR	2000 IU/mL	Plasma, serum	[74]
Gold nanoparticles	DNA-coated AuNP	HBsAg	Direct detection of SPR peak	0.1 IU/mL	Blood, serum, plasma	[26]
	DNA-coated AuNP	HBV DNA	Voltammetry	2 × 10^−9^ M	PCR product	[37]
	Anti-HBs and HAT-coated AuNP	HBsAg	FELISA	5 × 10^−4^ IU/mL	HBsAg in PBS	
	Oligo-coated AuNP	DNA	Colorimetric, disposable paper strips	1 × 10^−9^ M	N.A.	[27]
	Oligo-coated AuNP	HBV DNA	Colorimetric, dark-field microscope	1 × 10^−13^ M	PCR product	
		HBsAg	Electrochemical	0.343 pg/mL		[36]
	Gold Nanostructure	HBsAg	SERS	0.01 IU/mL	Serum	[46]
	Oligo-coated AuNP	HBV DNA	Electrochemical (impedance)	111 copies/mL	Serum	[35]
Magnetic nanoparticles	Immobilised, probe-conjungated NP	HBV DNA	Non-faradic impedance spectroscopy	50 pMol in 20 µL; 2.5 × 10^−6^ M	Plasma and serum	[63]
	Anti-HBsAg coated MNP	HBsAg	(cyclic) voltammetry	0.9 pg/mL	HBsAg in PBS	[60]
QDs	HBsAg-coated QDs	Anti-HBsAg	Lateral flow	2 pg/mL	Anti-HBsAg	[67]
Magnetite and gold nanoparticles	Immobilised gold NP, competition between target DNA and MNP	HBV DNA	R_CT_	3.1 (±0.1) × 10^−13^ M	Urine, plasma	[72]
	Anti-HBsAg coated MNP and AuNP aggregation	HBsAg	Anodic stripping voltammetry	87 pg/mL	HBsAg in PBS	[75]
AuNPs and QDs	Immobilised QD, competition between target DNA and AuNP	Simultaneous HBV DNA and HCV RNA	Colorimetric, ECL quenching	8.2 × 10^−14^ M (HBV) and 3.4 × 10^−13^ M (HCV)	Plasma	[76]

ELISA: enzyme-linked immunosorbent assay; HBeAg: hepatitis B virus e antigen; HBsAg: hepatitis B virus surface antigen; R_CT_: interfacial charge transfer resistance; QDs: quantum Dots; HAT: human alpha-thrombin; HCV: hepatitis C virus; SPR: surface plasmon resonance; FELISA: fluorescence enzyme-linked immunosorbent assay; SERS: surface enhanced Raman spectroscopy; ECL: electrochemiluminescence; PBS: phosphate-buffered saline; N.A.: non applicable.
